# STS1 and STS2 Phosphatase Inhibitor Baicalein Enhances the Expansion of Hematopoietic and Progenitor Stem Cells and Alleviates 5-Fluorouracil-Induced Myelosuppression

**DOI:** 10.3390/ijms24032987

**Published:** 2023-02-03

**Authors:** Na Li, Yanhong Wang, Anqing Wang, Jing Zhang, Chaoran Jia, Chunlei Yu, Zhenbo Song, Shuyue Wang, Lei Liu, Jingwen Yi, Yongli Bao, Yanxin Huang, Luguo Sun

**Affiliations:** 1 National Engineering Laboratory for Druggable Gene and Protein Screening, Northeast Normal University, Changchun 130024, China; 2 NMPA Key Laboratory for Quality of Cell and Gene Therapy Medicinal Products, Northeast Normal University, Changchun 130024, China; 3 The Cancer Institute, Qingdao University, Qingdao 266071, China; 4 Department of Medicine, Hematology/Oncology, Goethe University, 60590 Frankfurt, Germany

**Keywords:** STS1, STS2, baicalein, hematopoietic and progenitor stem cells, chemotherapy-induced myelosuppression

## Abstract

STS1 and STS2, as the protein phosphatases that dephosphorylate FLT3 and cKIT, negatively regulate the self-renewal and differentiation of hematopoietic stem and progenitor cells (HSPCs). To obtain the small molecule inhibitors of STS1/STS2 phosphatase activity used to expand HSPCs both in vitro and in vivo, we establish an in vitro phosphatase assay using the recombinant proteins of the STS1/STS2 histidine phosphatase (HP) domain, by which we screened out baicalein (BC) as one of the effective inhibitors targeting STS1 and STS2. Then, we further demonstrate the direct binding of BC with STS1/STS2 using molecular docking and capillary electrophoresis and verify that BC can restore the phosphorylation of FLT3 and cKIT from STS1/STS2 inhibition. In a short-term in vitro culture, BC promotes profound expansion and enhances the colony-forming capacity of both human and mouse HSPCs along with the elevation of phospho-FLT3 and phospho-cKIT levels. Likewise, in vivo administration with BC significantly increases the proportions of short-term hematopoietic stem cells (ST-HSCs), multipotent progenitors (MPPs) and especially long-term HSCs (LT-HSCs) in healthy mouse bone marrow and increases the numbers of colony-forming units (CFU) formed by HSPCs as well. More importantly, pre-administration of BC significantly enhances the survival of mice with lethal 5-fluorouracil (5-FU) injection due to the alleviation of 5-FU-induced myelosuppression, as evidenced by the recovery of bone marrow histologic injury, the increased proportions of LT-HSCs, ST-HSCs and MPPs, and enhanced colony-forming capacity. Collectively, our study not only suggests BC as one of the small molecule candidates to stimulate HSPC expansion both in vitro and in vivo when needed in either physiologic or pathologic conditions, but also supports STS1/STS2 as potential therapeutic drug targets for HSPC expansion and hematopoietic injury recovery.

## 1. Introduction

Hematopoietic stem cells (HSCs), characterized by self-renewal and multipotent differentiation capabilities, are crucial for both normal and stress-induced hematopoiesis [1]. HSCs have been widely used in clinic, such as hematopoietic stem cell transplantation (HSCT) for malignant hematological disease therapy. However, the limited number of hematopoietic stem and progenitor cells (HSPCs) may lead to delayed neutrophil engraftment, low efficiency of homing and reconstitution, and increased mortality [2,3]. Therefore, how to enhance the expansion and improve the self-renewal ability of HSCs has been a big challenge for researchers [4]. On the other hand, chemotherapy is a common modality used in cancer treatment, and chemotherapy-induced myelosuppression, which is a decrease in the ability of bone marrow to produce blood cells, causes many side effects, including anemia, infection due to neutropenia and bleeding due to thrombocytopenia [5,6,7]. Therefore, myelosuppression significantly affects the effectiveness of treatment and the patient’s quality of life, and even causes death. In clinic, hematopoietic drugs, such as some growth factors, are commonly used to treat myelosuppression, and concomitant chemotherapy needs to be delayed [8]. Enhancing the resistance of HSPCs to the damage caused by chemotherapy drugs is thus essential for the prevention of myelosuppression and the accomplishment of chemotherapy cycles. All the above circumstances underscore the importance of maintenance or enhancement of the number and function of HSCs. Therefore, it is necessary to obtain potent, yet specific, biological or chemical agents to efficiently expand HSCs and potentiate their functions.

The balance between self-renewal and multiple differentiation of HSCs depends on the regulation of growth factors, transcription factors, surface receptors, etc. [9]. FMS-like tyrosine kinase 3 (FLT3) and cKIT, which belong to the type III receptor tyrosine kinase (RTK) family, are expressed mainly in the hematopoietic compartment and play a key role in HSC self-renewal and early development [10,11,12]. Single FLT3 knockout mice are viable but display a defect in B lymphocyte progenitor cells, and mice lacking both FLT3 and cKIT display dramatic decrease in hematopoietic cells and higher mortality [13]. As cell surface receptors, ligand binding induces homodimerization and activation of the intrinsic tyrosine kinase activities of FLT3 and cKIT, which leads to their autophosphorylation and the initiation of several signal transduction cascades [14]. Therefore, dephosphorylation mediated by phosphatases negatively regulates the activities of FLT3 and cKIT and may also negatively modulate the proliferation and differentiation of HSPCs.

STS1 and STS2 are members of the suppressor of T cell receptor (TCR) signaling (STS) family [15], which are initially designated as negative regulators of TCR to ensure that T cells are not inappropriately activated. STS1 and STS2 are highly homologous, and both contain a ubiquitin-associated domain (UBA) in the N-terminus, an Src homology domain (SH3) and a C-terminal histidine phosphatase (HP) domain [16,17,18]. The HP domain, which is homologous to the phosphoglycerate mutase-like domain (PGM), has been shown to have robust tyrosine phosphatase activity [19,20] that can dephosphorylate numerous RTK proteins [19,21,22]. A recent study confirmed that STS1 and STS2 are the direct phosphatase of FLT3 and cKIT, and loss of STS1/STS2 induces hyperphosphorylation of FLT3, indicating that STS1/STS2 are the negative regulatory factors of FLT3 and cKIT signaling via dephosphorylation [15]. Accordingly, STS1/STS2 double knockout (dKO) mice show a significant hyperphosphorylation of FLT3 and cKIT and faster ex vivo proliferation of Lineage^−^Sca1^+^cKIT^+^ (LSK) cells. Moreover, STS1/STS2-deficient mice exhibit profound expansion of multipotent progenitor cells (MPPs), increased colony-forming ability and enhanced long-term hematopoietic reconstitution, implying an essential role of STS1/STS2 in regulating HSPC fitness, via negatively regulating FLT3 or cKIT [15]. Therefore, the pharmacological inhibition of STS1/STS2 phosphatase activity may provide a potential strategy for expansion of HSPCs both in vitro and in vivo.

In this study, we use the STS1/STS2 HP domain (hereinafter referred as STS1 or STS2) to establish its in vitro phosphatase assay, by which we screen effective small molecules that inhibit the phosphatase activities of STS1/STS2. Then, baicalein (BC), the main active ingredient of the *Scutellaria* root, is shown to be an effective phosphatase inhibitor of STS1/STS2 and abolishes the dephosphorylation of STS1/STS2 on FLT3 and cKIT. We then demonstrate that BC can enhance the hematopoietic properties of human and mouse HSPCs in vitro and in vivo, and, more importantly, BC elicits protective effects on 5-fluorouracil (5-FU)-induced myelosuppression in mice. Taken together, our findings support STS1/STS2 as targets for improving hematopoietic function and suggest BC as one small molecule candidate promoting hematopoiesis.

## 2. Results

### 2.1. Screening STS1 and STS2 Phosphatase Inhibitors

**Figure 1:**
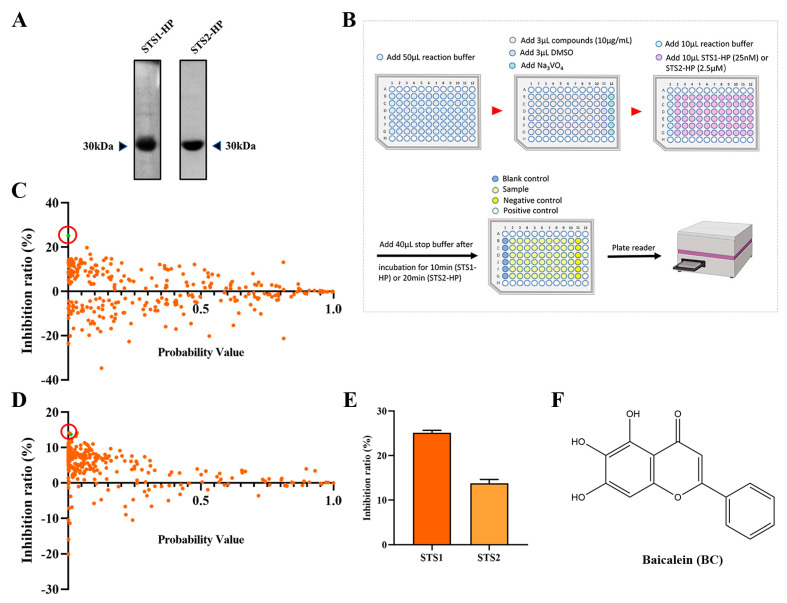
Screening STS1 and STS2 phosphatase inhibitors from natural small molecules. (**A**) Expression and purification of STS1 and STS2 histidine phosphatase (HP) domain recombinant proteins. Purified proteins were analyzed in 12% SDS−PAGE followed by Coomassie blue staining. (**B**) Schematic diagram of STS1 and STS2 phosphatase inhibitor screening. The reaction was carried out at room temperature. (**C**,**D**) Inhibition ratios of compounds (10 μg/mL) on STS1 (**C**) and STS2 (**D**) phosphatase activities. Probability values (*p*-value) indicate statistical significance. The green dot in the red circle represents baicalein (BC). (**E**) Inhibition ratios of BC (10 μg/mL) on STS1 and STS2 phosphatase activities (*n* = 6). The data represent the means ± SEM. (**F**) The chemical structure of BC.

As STS1 and STS2 act as phosphatases and play an important role in regulating hematopoietic stem and progenitor cell fitness, we used *p*-nitrophenyl phosphate (pNPP) as the substrate to establish an in vitro phosphatase assay with the aim of screening small molecular compounds that can inhibit the phosphatase activity of STS1/STS2. Recombinant human STS1 and STS2 proteins were expressed using the prokaryotic expression system and purified by Ni-NAT affinity chromatography (Figure 1A). We then determined the phosphatase activities of the recombinant STS1 and STS2 through the optimized phosphatase assay and obtained the enzyme kinetic parameters (Table 1). The enzyme kinetics of STS1 and STS2 were consistent with the previous study [20], indicating the good phosphatase activities of recombinant STS1/STS2. Comparatively, the recombinant STS2 exhibited lower K_m_ and k_cat_ values than STS1, which validates that STS2 has weaker phosphatase activity than STS1 [23]. Based on the established phosphatase assay, we screened a natural compound library consisting of 292 natural occurring small molecules (Figure 1B), and 10 μg/mL was used as the initial concentration of small molecules for screening, which is the commonly used concentration for drug screening with the compounds in the Chinese medicine monomer compound library of our laboratory for both in vitro and cell-based screening. Among 292 natural compounds, we found that 53 compounds (*p* < 0.05) could inhibit the phosphatase activity of STS1, and 103 compounds could inhibit the phosphatase activity of STS2 (Figure 1C,D). The compounds with an inhibition ratio higher than 20% were defined as effective STS1/STS2 phosphatase inhibitor candidates. We found that baicalin (BC) could significantly inhibit the phosphatase activity of STS1 in vitro (Figure 1C). However, no compounds exhibited significant phosphatase inhibitory activity against STS2 (Figure 1D), which might be related to its own weak phosphatase activity. Then we found that BC also inhibited STS2 phosphatase activity to some extent, but the inhibition ratio (13.77%) was lower than 20%. We finally chose BC as one of the STS inhibitor candidates to be further explored. The molecular structure of BC is shown in Figure 1F.

### 2.2. Validation of BC as a Phosphatase Inhibitor of STS1 and STS2

**Figure 2:**
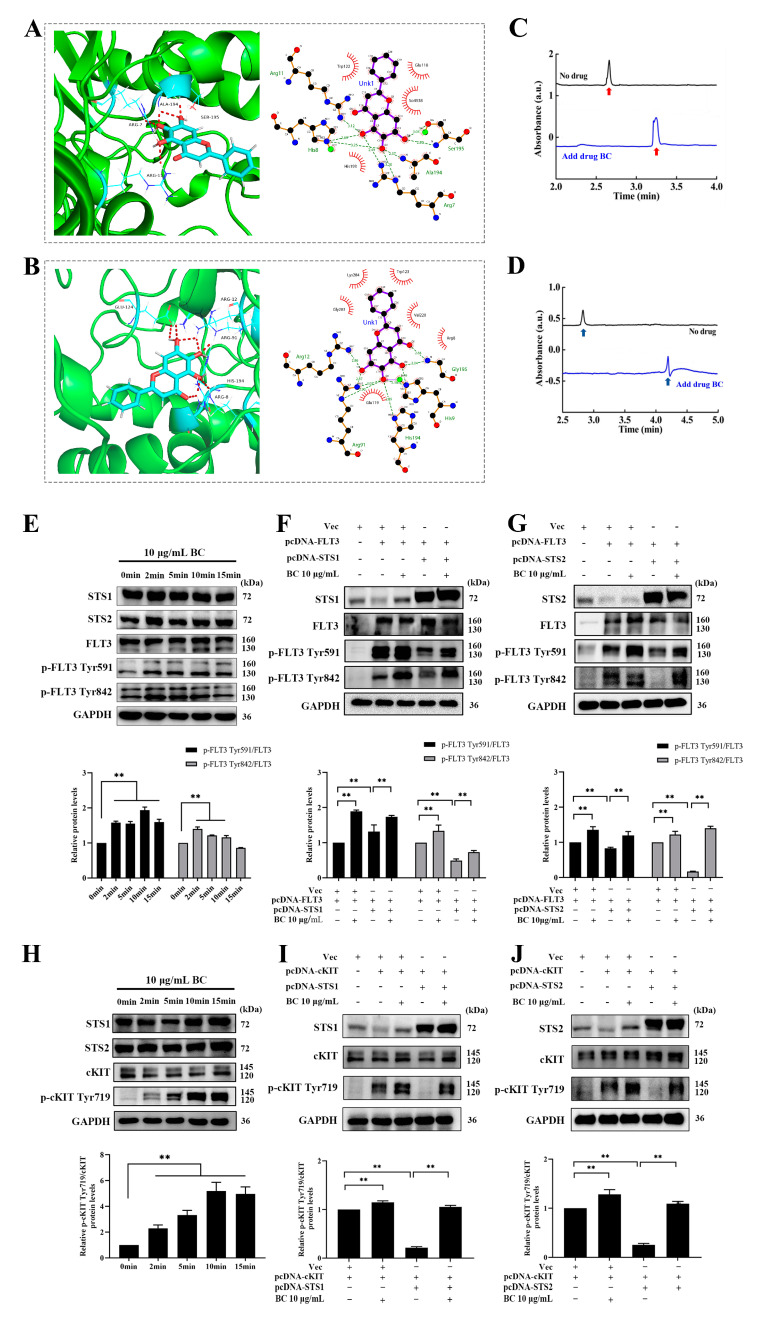
BC interacts with STS1 and STS2 HP domains and promotes phosphorylation of FLT3 and cKIT by inhibiting STS1 and STS2. (**A**,**B**) Molecular docking analysis of the binding of BC with STS1 (**A**) or STS2 (**B**). Left: The representative docking pose of BC with the HP domain STS1 (5VR6) or STS2 (5WDI). BC and the key residues of protein receptor are shown as sticks. The red dashed lines indicate the hydrogen bonds formed. Right: The 2D docked conformation showing the hydrophobic contacts (red eyelash shape) between BC and the HP domain of STS1 or STS2. (**C**,**D**) Capillary electrophoresis analysis of the binding of BC to STS1 (**C**) or STS2 (**D**). BC, STS1 or STS2 samples were prepared in 20 mM PBS at pH 7.0, and electrophoresis was run under 20 kV voltage. Red (**C**) or blue (**D**) arrows represent STS1 or STS2 protein peaks, respectively. (**E**,**H**) HEK293T cells were transiently transfected with FLT3 (**E**) or cKIT (**H**) constructs, and 48 h later, cells were stimulated with 10 μg/mL BC for different periods of time at 37 °C. Phosphorylated FLT3 (p−FLT3) or cKIT (p−cKIT) and endogenous STS1/STS2 proteins were detected by Western blot (*n* = 3). GAPDH was used as internal control. (**F**,**G**) FLT3 construct was transfected into HEK293T cells together with or without pcDNA−STS1 (**F**) or −STS2 (**G**). Then, 48 h later, cells were stimulated with BC for 2 min at 37 °C. The overexpressed proteins and phosphorylated FLT3 were detected by Western blot (*n* = 3). Vec indicates empty vector control. (**I**,**J**) cKIT construct was transfected into HEK293T cells together with or without Flag−tagged STS1 (**I**) or STS2 (**J**). Then, 48 h later, cells were stimulated with BC for 2 min at 37 °C. The overexpressed proteins and phosphorylated cKIT were detected by Western blot (*n* = 3). Vec indicates empty vector control. All data are shown as mean ± SEM, ** *p* < 0.01 (Student’s *t* test).

To explore whether BC directly binds to STS1/STS2 to inhibit their phosphatase activities, we performed molecular docking and capillary electrophoresis experiments. First, molecular docking of BC against STS1 or STS2 was carried out by using GOLD 5.2 software. Goldscore and ASP scores were used as the main scoring function and the repeated scoring function, respectively, and we defined Goldscore values higher than 50 as the presence of possible binding. As shown in Table 2, the Goldscores of BC with STS1 and STS2 were 51.64 and 54.33, respectively, both higher than 50. In addition, the docking poses showed the interaction between BC and the residues of STS1 or STS2 (Figure 2A,B). We observed that BC formed hydrogen bonds with STS1 residues Ala194, Arg7, Ser195 and Arg11 and had hydrophobic interactions with residues Trp122, Glu118, So4538 and His193 (Figure 2A). The same hydrogen bonds and hydrophobic interactions were also observed in the docking of BC against STS2, with hydrogen bond formation with residues Arg12, Glu124, Arg91, His194 and Arg8 and hydrophobic interactions with residues Lys284, Trp123, Gly283, Val1220, Arg8 and Glu119 (Figure 2B). Then, to validate the in silico docking results, we performed capillary electrophoresis experiments to detect the direct binding of BC with STS1 and STS2 subsequently. As shown in Figure 2C,D, the migration times of STS1 and STS2 proteins were markedly delayed when BC was added into the buffer, suggesting that BC has good binding with STS1 and STS2. Interestingly, although BC showed a lower phosphatase inhibition ratio to STS2 than to STS1 (Figure 1E), its binding to STS2 might be stronger than to STS1, as the migration time of the STS2 protein peak delayed further behind than STS1 when BC was added.

Next, we further examined whether BC inhibited the phosphatase activities of STS1/STS2 in cells. A previous study has reported that FLT3 and cKIT are the direct substrates of STS1/STS2, implying that inhibition of the phosphatase activity of STS1/STS2 can lead to hyperphosphorylation of FLT3 and cKIT [15]. First, we ectopically overexpressed FLT3 or cKIT in HEK293T cells, which were then treated with BC (10 μg/mL) for different periods of time. As we expected, the levels of phosphorylated FLT3 (at both Tyr591 and 842 sites) (Figure 2E) or cKIT (Figure 2H) were significantly increased upon BC treatment. Simultaneously, endogenous STS1 and STS2 were both shown to express in HEK293T cells to a different extent. Then we further co-overexpressed FLT3 or cKIT together with STS1 or STS2 in HEK293T cells, followed by BC treatment. We observed that the levels of phosphorylated FLT3 (Figure 2F,G) or cKIT (Figure 2I,J) were decreased when coexpressed with STS1 (Figure 2F,I) or STS2 (Figure 2G,J) compared with FLT3 or cKIT overexpression alone. This indicates that ectopically expressed STS1 or STS2 effectively dephosphorylated FLT3 or cKIT in HEK293T cells. However, BC treatment almost completely recovered the phosphorylation of FLT3 (Figure 2F,G) or cKIT (Figure 2I,J) from the inhibition by STS1 or STS2 co-expression. Thus, these results demonstrate that BC can inhibit the phosphatase activities of STS1 and STS2 in the cells, at least partly by which BC subsequently can enhance the phosphorylation of FLT3 and cKIT.

Taken together, BC may act as a phosphatase inhibitor of STS1 and STS2.

### 2.3. BC Treatment Promotes the Proliferation of Mouse BMMNCs and the Expansion of HPSCs In Vitro

**Figure 3:**
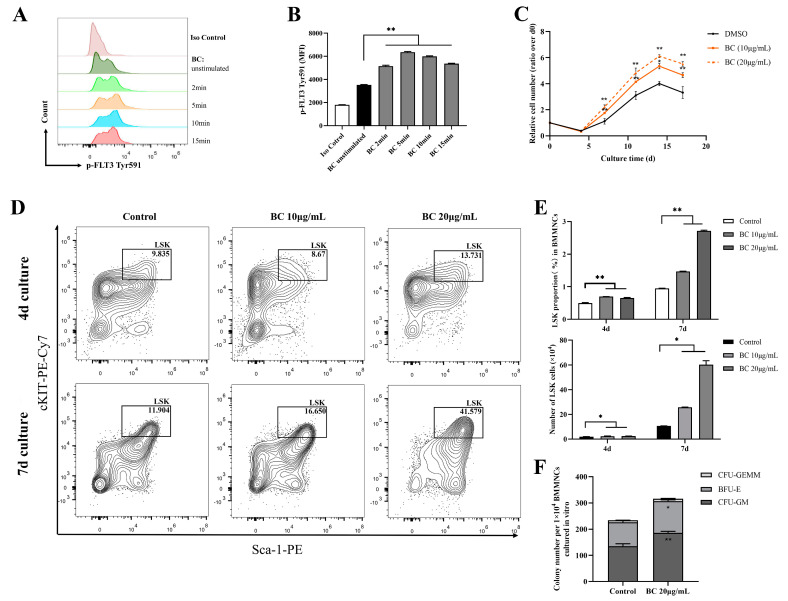
BC promotes the proliferation of mouse BMMNCs and the expansion of their HSPC population in vitro. (**A**,**B**) Flow cytometry analysis of FLT3 phosphorylation in mouse BMMNCs cultured in vitro with or without BC (10 μg/mL) treatment for 5–15 min. (**A**) Flow cytometry histogram showing the intensity of p−FLT3 Tyr591 versus cell counts. (**B**) The mean fluorescence intensity (MFI) of p−FLT3 Tyr591 from three independent experiments. (**C**) Cell counting of mouse BMMNCs cultured in vitro with or without BC treatment at different timepoints, which was presented as relative cell number to the cell number at day 0. Data represent three independent experiments. (**D**) Representative flow cytometry profiles to show the percentages of LSK (Lineage^−^Sca-1^+^cKIT^+^) in mouse BMMNCs cultured in vitro with or without BC treatment for 4 days or 7 days. Gates shown identify the LSK populations from Lineage^−^ (Lineage negative cells) population. (**E**) The proportion and absolute number of LSK population in (**D**). Data represent three independent experiments. (**F**) Colony counting of HSPCs derived CFUs from 1 × 10^4^ BMMNCs cultured in vitro with or without BC treatment for 4 days. CFU−GM: colony-forming unit of granulocyte and macrophage; BFU-E: burst−forming unit−erythroid; CFU−GEMM: colony−forming unit of granulocyte, erythrocyte, monocyte and megakaryocyte. Data represent triplicate experiments. All data are shown as mean ± SEM, * *p* < 0.05, ** *p* < 0.01 (Student’s *t* test).

FLT3 and cKIT are expressed in hematopoietic cells and are essential for the self-renewal and differentiation of HSPCs; therefore, they play key roles in the hematopoiesis process [24,25,26]. As the above data showed that BC had the potential to activate FLT3 and cKIT via inhibiting the activities of STS1 and STS2, we next intended to investigate whether BC had any beneficial impact on HSPCs. To this end, bone marrow mononuclear cells (BMMNCs) were isolated from the C57BL/6 mouse and were treated by BC in an in vitro culture. First, the effects of BC on the phosphorylation of FLT3 and cKIT in BMMNCs were assessed by flow cytometry. We used flow cytometry but not Western blot here because only a very small proportion of BMMNCs are HSPCs expressing FLT3 and cKIT, and we failed to detect p-FLT3 or p-cKIT in Western blot. Flow cytometry analysis using anti-p-FLT3 Tyr591 antibody showed that upon BC treatment for 2–15 min, the mean fluorescence intensity (MFI) was dramatically increased in BMMNCs (Figure 3A,B) compared to the untreated control, indicating the elevation of the p-FLT3 Tyr591 level by BC. For anti-p-FLT3 Tyr842 or anti-p-cKIT Tyr719 detection, the MFI was also increased to a different degree upon BC treatment, although the overall intensity was very low, perhaps because the antibodies may not be suitable for a flow cytometry assay (Figure S1A–D). These results suggest that BC may activate FLT3 or cKIT in mouse BMMNCs in vitro. Then, the mouse BMMNCs were cultured in the presence of 10 μg/mL or 20 μg/mL BC for up to 17 days in vitro. We found that the cell numbers of BC-treated groups were significantly dose-dependently higher than the control group (DMSO) at each time point, suggesting that BC induced a faster proliferation of BMMNCs (Figure 3C). The BMMNCs are a heterogeneous cell population, so we further performed a phenotypic analysis of HSPCs via flow cytometry using multiple marker combination. The results showed that the Lineage^−^Sca1^+^cKIT^+^ (LSK) cell population in BMMNCs cultured with BC for 4 days or 7 days was dose-dependently increased to a remarkable degree (Figure 3D,E). These data indicate that BC led to HSPC proliferation over 7 days of culture, and 20 μg/mL BC might be a more optimal dose (Figure 3E). To explore the impact of BC on the function of HSPCs cultured in vitro, BMMNCs treated with 20 μg/mL BC after 7 days were used to perform the CFU assay. As shown in Figure 3F, we found a significant increase in the total colony number in the BC-treated group compared with the control group. Morphological classification of multipotent progenitor-cell-derived colonies showed a comparable increase in colony numbers of granulocyte and macrophage (CFU-GM) and burst-forming unit-erythroid (BFU-E), indirectly reflecting that there was no lineage skewing of hematopoietic progenitor cells (HPCs) after BC treatment (Figure 3F). The number of granulocyte-erythrocyte-monocyte-megakaryocyte colonies (CFU-GEMM) also had a slight increase compared with the control group but did not reach statistical significance (Figure 3F). Collectively, the above results demonstrate that BC can enhance the expansion and short-term self-renewal capacity of mouse HSPCs cultured in vitro, and activation of FLT3 and cKIT by BC through inhibiting STS1/STS2 may be one of the underlying mechanisms.

### 2.4. BC Treatment Enhances the In Vitro Expansion of Primitive HSCs and the Multipotency of HSPCs in Human CD34^+^ Umbilical Cord Blood Cells

**Figure 4:**
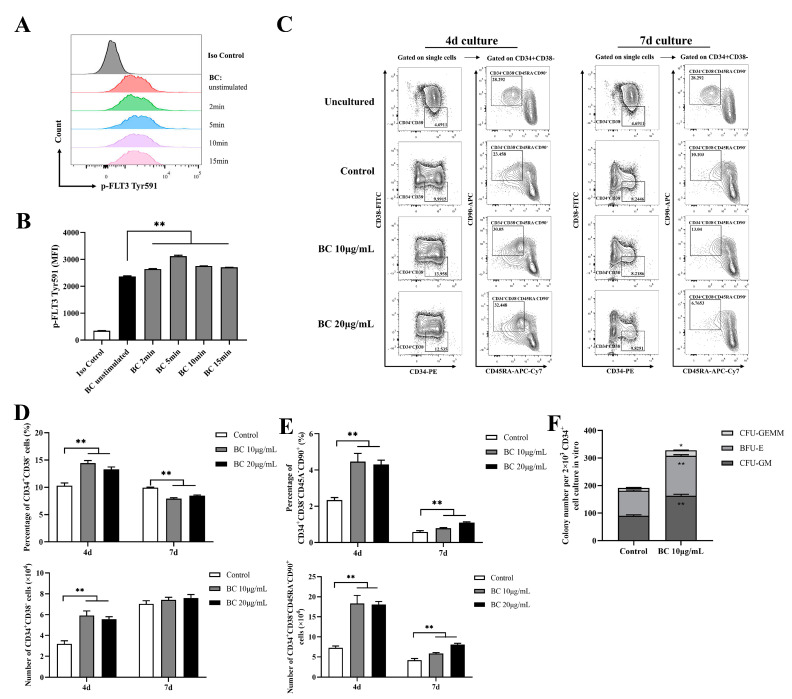
BC increases the proportion of primitive HSC population and colony−forming ability of human CD34^+^ cord blood cells in vitro. (**A**,**B**) Flow cytometry analysis of FLT3 phosphorylation in CD34^+^ UCB cells cultured in vitro with or without BC (10 μg/mL) treatment for 2–15 min. (**A**) Flow cytometry histogram showing the intensity of p−FLT3 Tyr591 versus cell counts. (**B**) The mean fluorescence intensity (MFI) analysis of p−FLT3 Tyr591 from three independent experiments. (**C**) Representative flow cytometry profiles to show the percentage of CD34^+^CD38^−^ and CD34^+^CD38^−^CD45RA^−^CD90^+^ cells in CD34^+^ UCB cells cultured in vitro with or without BC treatment for 4 days or 7 days. Gates shown identify the CD34^+^CD38^−^ and CD34^+^CD38^−^CD45RA^−^CD90^+^ populations from CD34^+^ cells. (**D**,**E**) The proportion and absolute number of CD34^+^CD38^−^ and CD34^+^CD38^−^CD45RA^−^CD90^+^ cells in (**C**). Data represent three independent experiments. (**F**) Colony counting of HSPCs derived CFUs from 2 × 10^3^ CD34^+^ UCB cells cultured in vitro with or without BC treatment for 7 days. Data represent triplicate experiments. All data error bars are shown as mean ± SEM, * *p* < 0.05, ** *p* < 0.01 (Student’s *t* test).

Given that BC significantly improved the function of mouse HSPCs cultured in vitro, we examined whether it has a similar effect on human HSPCs. We obtained CD34^+^ cells from fresh human umbilical cord blood (UCB), and likewise first tested the effect of BC on phosphorylation of FLT3 and cKIT in CD34^+^ UCB cells using flow cytometry analysis. In accordance with the results shown in mouse BMMNCs, BC treatment for 2–15 min led to a general significant increase in the MFI of p-FLT3 Tyr591 staining in CD34^+^ UCB cells (Figure 4A,B), and a similar increasing trend was also observed in the MFI of p-FLT3 Tyr842 and p-cKIT Tyr719 staining, but the MFI value was still very low (Figure S2A–D). This result suggests that BC may also activate FLT3 and cKIT in human HSPCs. HSPCs contain both MPPs and HSCs; MPPs are responsible for quickly replenishing blood cells under stress-induced hematopoiesis, while HSCs possess self-renewal ability, and an increase in the proportion of HSCs indicates the enhancement of long-term hematopoietic reconstitution ability [27]. Thus, the HSPC and HSC populations were both assessed in this part. Although CD34 is commonly used to identify human hematopoietic stem cells, CD34^+^ cells are a heterogeneous population including hematopoietic stem cells and progenitor stem cells [28,29]. Thus, CD38, CD45RA and CD90 are further used to distinguish different subpopulations, which defines CD34^+^CD38^−^ as HSPCs and CD34^+^CD38^−^CD45RA^−^CD90^+^ as HSCs [29,30]. We found that when CD34^+^ UCB cells were cultured in vitro for 4 days, there was a considerable increase in the proportions and absolute number of both the CD34^+^CD38^−^ (HSPCs) population (Figure 4C,D) and CD34^+^CD38^−^CD45RA^−^CD90^+^ (HSCs) (Figure 4C,E) subpopulations upon BC treatment in comparison with the control group, but 10 μg/mL BC was more effective than 20 μg/mL. In contrast, for the 7-day culture, BC treatment only significantly increased the proportion and absolute number of the CD34^+^CD38^−^CD45RA^−^CD90^+^ (HSCs) subpopulation (Figure 4C,E) compared with the untreated control group. However, the proportion of the CD34^+^CD38^−^ (HSPCs) population cultured with BC was lower than the control group (Figure 4C,D), while the absolute number of CD34^+^CD38^−^ cells had little increase compared with the control group (and did not reach statistical significance). Finally, we performed the CFU assay with human CD34^+^ UCB cells which were cultured in vitro with 10 μg/mL BC for 4 days. As expected, BC-treated cells displayed higher numbers of three types of colonies (CFU-GM, BFU-E and CFU-GEMM) as well as total colonies than control cells (Figure 4F). These data demonstrate that BC can support the in vitro expansion of human HSCs and enhance their multipotentiality and HSPCs at least partly via activating FLT3 and cKIT.

### 2.5. In Vivo Administration of BC Increases the Proportion and Multilineage Differentiation Potential of Healthy Mouse Bone Marrow HSPCs

**Figure 5:**
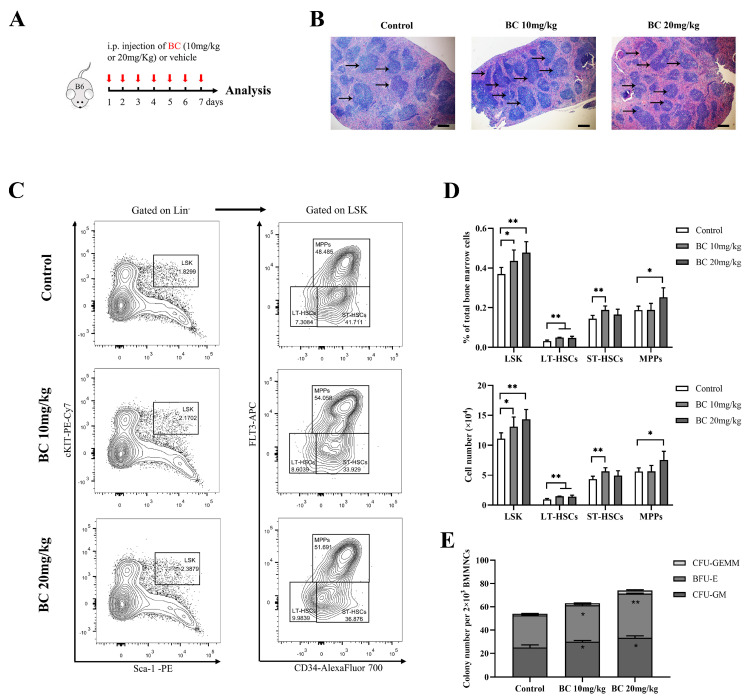
BC administration increases the proportion of HSPC population and colony−forming ability of BMMNCs in healthy mice. (**A**) Experimental schematic of BC administration in healthy mice. C57BL/6 mice were administered once daily with BC (10 or 20 mg/kg) or vehicle by intraperitoneal (i.p.) injection for 7 days (*n* = 5). Mice were sacrificed 24 h after the last injection. B6 represents C57BL/6 mice. Red arrows indicate BC injection timepoints. (**B**) Histological analysis of spleen tissues by hematoxylin and eosin (HE) staining. Scale bar: 200 μm. Black arrows indicate the white pulp of spleen. (**C**) Representative flow cytometry profiles showing the percentage of LSK (left), LT−HSCs (LSKCD34^−^FLT3^−^), ST-HSCs (LSKCD34^+^FLT3^−^) and MPPs (LSKCD34^+^FLT3^+^) (right) in BMMNCs from control or BC (10 or 20 mg/kg) treated mice. Gates shown identify the LSK populations from Lineage^−^ (Lineage negative cells) and the LT−HSC, ST−HSC and MPP populations from LSK cells. (**D**) The proportions and absolute number of LSK, LT−HSCs, ST−HSCs and MPPs in BMMNCs from control or BC (10 or 20 mg/kg) treated mice in flow cytometry analysis as shown in (**C**) (*n* = 5). (**E**) The numbers of CFUs formed by BMMNCs from control or BC (10 or 20 mg/kg) treated mice (*n* = 3). All data are shown as mean ± SEM, * *p* < 0.05, ** *p* < 0.01 (Student’s *t* test) versus control.

The above results demonstrated the enhancement effect of BC on the proliferation and differentiation of both mouse and human HSPCs in vitro; next, we intended to explore the functional roles of BC on hematopoiesis in vivo. C57BL/6 mice were dosed interperitoneally (i.p.) once daily with BC (10 and 20 mg/kg) or vehicle for 7 days and were then subjected to multiple examinations 24 h after the last dosing (Figure 5A). We noticed that 7 consecutive days of BC administration at either dose did not significantly affect the body weight (Figure S3A), hematological parameters (Figure S3B–D) or spleen index (Figure S3E) of mice. Meanwhile, histological analysis of livers and kidneys also showed no pathological changes (Figure S3F–I). Therefore, we believe that short-term i.p. injection of BC has no obvious toxic effects on mice.

Notably, histological examination of the spleen displayed an increased amount of white pulp and germinal centers in mice with BC administration (Figure 5B), suggesting the enhanced proliferation and differentiation of T and B-lymphocytes [31]. In addition, BMMMNCs were isolated from mice and labeled for designated antibodies to analyze the phenotype of HSPCs by flow cytometry. As displayed in Figure 5C,D, the proportion of LSK showed a significant dose-dependent increase in mice injected with BC. Within the LSK population, the proportion of LT-HSCs markedly increased in both doses of BC-treated mice, while the proportion of ST-HSCs or MPPs significantly increased only in 10 mg/kg or 20 mg/kg BC-treated mice, respectively (Figure 5D). Interestingly, although BC led to the increase in MPP and HSC populations of mouse BM, the number of peripheral blood cells did not increase significantly (Figure S3B–D). We speculate that healthy mice have a self-adjusting ability to keep a steady state of peripheral blood cell levels, and BC might enhance the self-repairing capability of bone marrow that has been injured. In addition, the CFU assay showed that the number of total colonies and the three types of colonies (CFU-GM, BFU-E and CFU-GEMM) formed by HSPCs in BMMNCs were all obviously increased in BC-treated mice in a dose-dependent manner (Figure 5E), which was consistent with the in vitro results (Figure 3F). Overall, these data indicate that BC can potentiate hematopoiesis of healthy mice by elevating the proportion of HSPCs, especially LT-HSCs, and may also improve the immunity of mice.

### 2.6. BC Pretreatment Improves the Survival and Ameliorates Myelosuppression in 5-FU-Injured Mice

**Figure 6:**
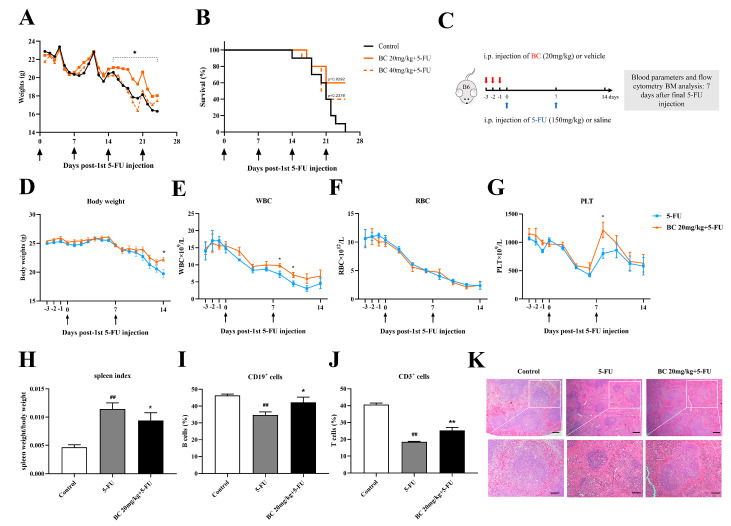
BC pretreatment enhances survival and ameliorates myelosuppression in mice with cycles of 5−FU injection. (**A**,**B**) Body weight and survival of mice that were i.p. injected with vehicle or BC (20 or 40 mg/kg) for 3 days (once a day) before being repeatedly injected with 5−FU (150 mg/kg) once a week (*n* = 10). Arrows indicate the time points of injection of 5-FU. (**C**) Experimental schematic of evaluating the effects of BC pretreatment on 5−FU−injured bone marrow in mice. B6 represents C57BL/6 mice. Red arrows indicate BC injection timepoints. (**D**) Mouse body weights (*n* = 5). (**E**−**G**) WBC (**E**), RBC (**F**) and PLT counts (**G**) of mouse peripheral blood measured every 2 days (*n* = 5). Arrows indicate the time points of injection of 5-FU. (**H**−**J**) Mouse spleen index (**H**) and flowcytometry analysis of the CD19^+^ (**I**) and CD3^+^ cells (**J**) in mouse spleen (*n* = 5). Spleens were removed at day 7 after the second cycle of 5−FU injection. (**K**) Histopathological analysis of spleen tissue by HE staining. Scale bars: 200 μm (upper panel) and 100 μm (lower panel). All data are shown as mean ± SEM. ## *p* < 0.01, compared with control group. * *p* < 0.05, ** *p* < 0.01, compared with 5-FU group (Student’s *t* test).

Acute stressors such as chemoradiotherapy usually cause hematopoietic toxicity and even HSC exhaustion [32]. As in vivo administration of BC can promote HSPC function in healthy mice, we reasonably speculate whether BC elicits protective effects in mice with BM injury under stress conditions. To this end, we established a myelosuppression mouse model by using 5-FU, a chemotherapeutic drug that has a toxic effect on rapidly proliferating cells [33]. Mice were i.p. injected with a lethal dose of 5-FU (150 mg/kg), a common dose used in establishing a hematopoietic injury model [34,35,36], once a week for 4 weeks, and then a series of preventive and therapeutic dosage regimens were tested to evaluate the effects of BC on the survival of mice with myelosuppression and HSC exhaustion (Figure S4). We found that BC (10 and 20 mg/kg) pretreatment for 3 consecutive days before the first 5-FU injection significantly enhanced the survival of 5-FU-injured mice. What was unexpected, mice injected with BC and 5-FU simultaneously or injected with BC 24 h after the first 5-FU injection exhibited no marked improvement in survival (Figure S4). As 20 mg/kg pretreatment showed better protective effects than 10 mg/kg, we speculate whether higher doses of BC may have even better protective effects. Therefore, we administered higher doses of BC (20 and 40 mg/kg) to mice for 3 consecutive days before 5-FU injection to further investigate the improving effect of BC on the survival of 5-FU-induced hematopoietic injury mice. We found that both doses of BC pretreatment alleviated the loss of body weight of mice after the second round of 5-FU injection (Figure 6A) and, similarly, improved the survival of mice from 0 to 40–60% (Figure 6B). Moreover, 20 mg/kg BC elicited better protective effects than 40 mg/kg; thus, 20 mg/kg was used in subsequent experiments.

**Figure 7:**
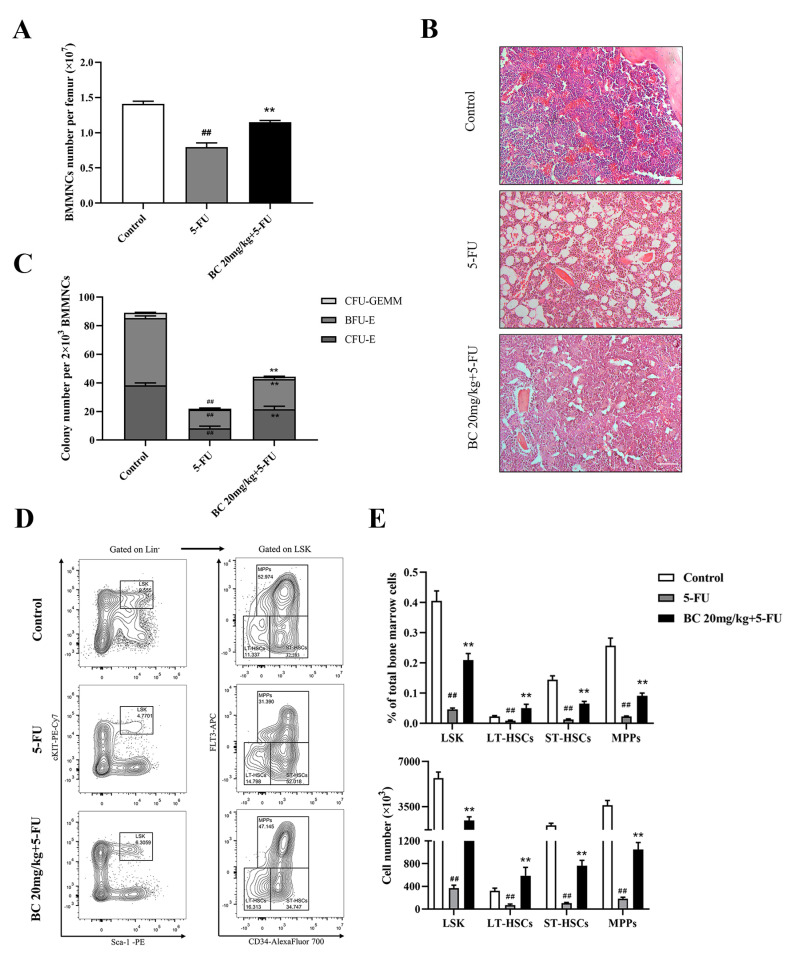
BC pretreatment protects bone marrow hematopoietic stem and progenitor cells from 5−FU injury in mice. (**A**) Counting of the total cells in BM isolated from unilateral femur of mice treated as shown in Figure 6C (*n* = 5). (**B**) Histopathological analysis of bone marrow from unilateral femur of mice treated as shown in Figure 6C by HE staining. Scale bar: 100 μm. (**C**) CFU assay of BM cells isolated from unilateral femur of mice treated as shown in Figure 6C (*n* = 3). (**D**) Representative flow cytometry profiles showing the proportions of LSK (left), LT−HSCs, ST−HSCs and MPPs (right) in BM of mice treated as shown in Figure 6C (*n* = 5). Gates shown identify the LSK populations from Lineage^−^ (Lineage negative cells) population and the LT−HSCs, ST−HSCs and MPPs subpopulations from LSK population. (**E**) The proportions and absolute numbers of LSK cells, LT−HSCs, ST−HSCs and MPPs in BMMNCs based on flow cytometry analysis shown in (**D**) (*n* = 5). All data are shown as mean ± SEM. ## *p* < 0.01, compared with control group. ** *p* < 0.01, compared with 5-FU group (Student’s *t* test).

We next investigated the effects of BC on bone marrow hematopoietic function of 5-FU-injured mice (Figure 6C–K and Figure 7). Mice were again pretreated with 20 mg/kg BC or vehicle for 3 consecutive days followed by an injection of a lethal dose of 5-FU once a week, but they were sacrificed 7 days after the second cycle of 5-FU injection (Figure 6C) and subjected to designated evaluations. Besides ameliorating the loss of body weight as shown in Figure 6A (Figure 6D), BC pretreatment also improved the number of white blood cells (WBC) and platelets (PLT) but not red blood cells (RBC) to different degrees in the peripheral blood of 5-FU-injured mice (Figure 6E–G). In addition, control mice with 2 cycles of 5-FU injection displayed an excessive increase in the spleen index (Figure 6H) but a decrease in the proportions of CD19^+^ B cells and CD3^+^ T cells in the spleen (Figure 6I,J), which were, however, all relieved to some extent by BC pretreatment (Figure 6H–J). Histological analysis of the spleen showed that 5-FU induced splenic injury as indicated by disturbed white and red pulp structures, smaller germinal centers and obscure marginal zone between red and white pulp, while BC dosing prior to 5-FU injection obviously improved the histological structure of the spleen (Figure 6K). All the above data demonstrate that prophylactic administration of BC can enhance the survival and ameliorate the myelosuppression of mice that underwent multiple rounds 5-FU injection.

### 2.7. BC Ameliorates 5-FU-Induced Impairment of HSCs and Progenitor Cells

We finally analyzed the effect of BC pretreatment on 5-FU-induced impairment on bone marrow in mice, by which BC protected mice from lethal 5-FU toxicity. Counting of the BMMNCs and histological analysis of the bone marrow showed that BC pretreatment partially restored the cell number of BMMNCs which were markedly decreased upon 5-FU treatment (Figure 7A) and cellularity (Figure 7B) of bone marrow in 5-FU-injured mice. Furthermore, the reduced colony-forming ability of HSPCs in the bone marrow of 5-FU-injured mice was also partially rescued by BC pretreatment (Figure 7C). Importantly, subsequent flow cytometry analysis showed that the proportion and absolute number of the LSK population and LT-HSC, ST-HSC and MPP subpopulations in BM were significantly increased upon BC pretreatment compared with 5-FU-injured control mice (Figure 7D,E), suggesting that BC pretreatment effectively protected HSPCs from 5-FU-induced loss. These findings indicate that prophylactic administration of BC provides protection against 5-FU-induced BM damage.

## 3. Discussion

Enhancing the expansion and self-renewal capacity of human HSPCs both in vitro and in vivo is essential for the success of clinical HSC transplantation and the resistance to myelosuppression. Here, we for the first time identify the natural compound BC as a potent STS1/STS2 phosphatase activity inhibitor by screening, and we further demonstrate that BC can significantly expand both mouse and human HSPCs in vitro. Above all, BC can also promote the expansion and activity of HSPCs in vivo and thus effectively protect mice against 5-FU-induced myelosuppression and death. Our study provides more evidence to support that STS1/STS2 can be used as drug targets for HSPC expansion and presents BC as a small molecule candidate to stimulate HSC expansion when needed.

STS1 and STS2 share 75% amino acid homology, but the phosphatase activity of STS1 is much stronger than that of STS2 [23,37], which was also observed when we measured the enzyme kinetic parameters of STS1 and STS2 using the in vitro phosphatase assay (Table 1). As STS1 has a higher phosphatase activity, we found that it is more efficient to screen out inhibitors on STS1, while no compounds were identified inhibiting STS2 with statistical significance during the initial screening from the same bank of natural compounds. However, the compound inhibitors of STS1 we identified, including BC, almost all exhibited suppressive effects on STS2 to some extent, although weaker than that on STS1 in the in vitro phosphatase assay (Figure 1C,D and unpublished data). Molecular docking and capillary electrophoresis assay demonstrated that BC could directly bind to both STS1 and STS2, which further supports that BC can act as a dual inhibitor on both STS1 and STS2. Interestingly, BC showed a stronger interaction with STS2 than with STS1 in capillary electrophoresis analysis (Figure 2B,D), which requires more assessment methods to confirm. STS1 has been shown to be widely expressed in tissues, while STS2 is more specifically expressed in the hematopoietic system, such as the thymus, spleen and bone marrow [38,39]. As STS1 and STS2 share high homology and the activity of STS1 is much higher than STS2, STS1 and STS2 seem functionally redundant especially in the hematopoietic system. However, the knockout of both STS1 and STS2 strengthened the expansion of LSK, the colony-forming ability and the hyperphosphorylation of FLT3 compared with the knockout of STS1 or STS2 alone [15]. Therefore, inhibition of STS2 activity is indispensable for HSC expansion. Dual inhibitors such as BC that can suppress both STS1 and STS2 would possess more advantages to stimulate HSC expansion.

FLT3 and cKIT are key regulators in the development and differentiation of HSPCs, and c-KIT ligand and FLT3 ligand have commonly been used in HSC cultures in vitro together with other cytokines. FLT3 and cKIT are direct substrates of phosphatases STS1 and STS2. As expected, BC restored the levels of phosphorylated FLT3 and cKIT which were suppressed by overexpressed STS1 or STS2 in HEK 293T cells. More importantly, BC induced the hyperphosphorylation of FLT3 and cKIT in mouse primary BMMNCs and human CD34^+^ UCB cells in vitro, indicating the inhibition of BC on cellular STS1 and STS2 in primary HSPCs. Accordingly, BC caused profound expansion of mouse LSK populations and human primary HSC populations and enhanced the capacity of HSPCs to differentiate into multilineages in vitro. Consistently, 7 consecutive days of BC administration in vivo led to the increased proportions of ST-HSCs, MPPs and especially the LT-HSC population in mouse BM and also enhanced the multilineage differentiation capacity of BM, indicating the enhanced short-term reconstituting ability. Surprisingly, we observed an increased area of white pulps and number of germinal centers in the spleens of BC-treated mice. It is well known that the white pulp contains the T-cell area and B-cell area [40], and the proliferation and differentiation of B-lymphocytes occurs in the germinal centers of the spleen [31]. Thus, this result indicates that BC administration may cause either more lymphocytes from BM into the spleen or enhanced B lymphocyte activity (producing antibodies) in situ, suggesting that BC may have the potential to improve the immunity of mice. Altogether, we speculate that the advantage and improvement of BC on HSPC expansion and function are at least partially related to the activation of FLT3 and cKIT by inhibiting STS1/STS2, which, however, would be verified if we could examine the effects of BC on HSPCs in STS1/STS2 knockout mice.

The competitive transplantation experiment is the gold standard to determine the long-term reconstitution capacity of HSCs in vivo. Regrettably, we could not obtain mice with the CD45.1 genetic background in China during these years of the epidemic and thus did not conduct this experiment. However, we did test the protective effects of BC on myelosuppression in mice. The most serious side effect of chemotherapy for the treatment of cancer is myelosuppression, and the survival of enough HSPCs is essential for the recovery of BM cellularity and mature blood cells [33,41]. We found that BC administration for 3 consecutive days before the first 5-FU injection can significantly enhance the survival of mice from multiple rounds of injection of lethal doses of 5-FU. 5-FU is cytotoxic to ST-HSCs and MPPs which are in the rapid proliferation phase; hence, LT-HSCs are crucial to compensate myeloablation [42]. Indeed, we found that 5-FU-injured mice with preadministration of BC had an obviously higher proportion of LT-HSCs in BMMNCs compared with 5-FU control mice (Figure 7E). Of note, BC administration concurrently with 5-FU or 24 h after the first 5-FU injection exhibited no protective effects on 5-FU-injured mice. This indicates that BC pretreatment may facilitate the storage of sufficient amounts of HSCs, especially LT-HSCs, which endows the mice with the resistance to lethal 5-FU injury to some extent, while once the injury of 5-FU on BM is initiated, BC may not stimulate enough HSCs at all. Moreover, we also observed the protective effect of BC on lethal radiation. Likewise, only pre-administration of BC before irradiation could obviously increase the survival of radiation-damaged mice. Actually, it has been reported that BC can mitigate radiation-induced hematopoietic injury either by rebalancing gut microbiota and inhibiting apoptosis [43] or through antioxidative damage via activating ERK and nuclear factor erythroid 2-related factor 2 (Nrf2) [44]. The crosstalk of BC inhibiting STS1/STS2 with the above recommended mechanisms, such as inhibiting apoptosis and activation of ERK and Nrf2, deserves further investigation. In addition, we cannot exclude the possibility that BC may have other hematopoiesis-related targets in vivo.

BC is one of the most representative flavonoid ingredients extracted from the root of *Scutellaria baicalensis Georgi,* the well-known traditional Chinese herb. BC has various pharmacological activities, including neuroprotection, antidepressant, anticancer, anticoagulation, free radical scavenging and antioxidant [45,46,47,48], and it has historically been used to treat cancer, infection and hepatic disorders in the clinic for many years [49].

Many studies have explored the in vivo processes of BC. After oral administration, BC is subjected to the first-pass effect in the liver and intestine [50,51] and in enterohepatic circulation [52]. Then BC is mainly distributed in the liver and lungs, as well as in the brain, eyes, thymus and other organs, and finally excreted by feces, bile and kidneys [53,54,55,56]. No obvious side effects of BC have been observed clinically or experimentally. For example, there were no serious adverse drug events after single oral doses of 100–2800 mg of BC or multiple oral administrations within the dose range of 200–800 mg of BC [55,57,58]. Therefore, the favorable safety profile ensures that further clinical studies on BC can be carried out. Baicalein aluminum capsules, the currently marketed baicalein-related drug, are used mainly to treat acute enteritis. Baicalein tablets are undergoing Phase I and II clinical trials to treat respiratory tract infection, influenza, tumors and sepsis.

It has been reported that BC has the ability to inhibit hematological malignancies; however, the drug targets of BC have never been elucidated, and the application of BC in stimulating hematogenesis and alleviating chemotherapy-induced myelosuppression has not been reported either. In Chinese traditional medicine, *Scutellaria baicalensis* is used to clear away heat and dampness, purge fire and detoxify the body [59], but we notice that there are a few reports by Russian scientists showing that administration of dry *Scutellarin baicalensis* extract stimulated hemopoiesis in lung cancer patients during antitumor chemotherapy [60] and produced a significant erythropoiesis-stimulating effect under the conditions of cytostatic myelosuppression [61]. This means that the enhancement effects of BC on HSPCs may account for the stimulating effects of *Scutellarin baicalensis* on hemopoiesis. As STS1 and STS2 can dephosphorylate many RTKs, such as epidermal growth factor receptor, platelet-derived growth factor receptor and so on [19,21,22], and as STS1 is widely expressed, we believe that at least some pharmacological activities of BC may contribute to targeting STS1/STS2. More activities and drug targets of BC require further exploration.

Taken together, our data demonstrate, for the first time, that BC acts as an inhibitor of STS1 and STS2 phosphatase activities and highlight the functional roles of BC to enhance the expansion of HSPCs both in vitro and in vivo, suggesting its potential application in hematopoietic recovery.

## 4. Materials and Methods

### 4.1. Reagents and Antibodies

In total, 292 natural monomeric compounds used in screening phosphatase inhibitors were extracted from traditional Chinese herbs and structurally clarified by the National Engineering Laboratory for Druggable Gene and Protein Screening of Northeast Normal University, and the purity of all the compounds surpassed 98%. Compounds were dissolved in dimethyl sulfoxide (DMSO) at 1 mg/mL and diluted with saline solution to 0.2 mg/mL as the working solution, which was stored at −20 °C. Baicalein (B20571) used in cell and animal experiments was purchased from Yuanye Bio-Technology (Shanghai, China), with a purity of over 98%.

Human stem cell factor (SCF) (#300-07), human thrombopoietin (TPO) (#300-18), human interleukin (IL)-6 (#200-06), murine SCF (#250-03), murine TPO (#315-14) and murine IL-3 (#213-13) were purchased from Peprotech (Rocky Hill, NJ, USA). Flow cytometric antibodies against mouse hematopoietic Lineage-FITC cocktails (#22-7770-72), mouse Sca-1-PE (#12-5981-82), mouse cKIT-PE-Cy7 (#25-1178-42), mouse FLT3-APC (#17-1351-82), mouse CD34-Alexa Fluor 700 (#56-0341-82), mouse CD3-FITC (#11-0032-82), mouse CD19-APC (#17-0193-82) and human CD45-APC (#17-0459-42) were purchased from eBioscience (San Diego, CA, USA). Human CD34-PE (#343506), human CD38-FITC (#980304), human CD45RA-PE-Cy7 (#983006) and human CD90-APC (#328114) were purchased from Biolegend (San Diego, CA, USA). Specific antibodies against FLT3 (#3462), phospho-FLT3 Tyr591 (#60413), phospho-FLT3 Tyr842 (#4577), cKIT (#3074) and phospho-cKIT Tyr719 (#3391) were obtained from Cell Signaling Technology (Danvers, MA, USA). Specific antibodies against STS1 (#19563), STS2 (#15823) and Flag (#80010) were obtained from Proteintech (Chicago, IL, USA).

### 4.2. Plasmids and Cell Lines

The pcDNA-FLT3 eukaryotic overexpression plasmids were described previously [15]. The pNIC28-Bsa4-STS1 and pNIC28-Bsa4-STS2-HP prokaryotic expression plasmids were synthesized by GenScript (Nanjing, China). The pcDNA-STS1 and pcDNA-STS2 eukaryotic expression plasmids were synthesized by GenScript (Nanjing, China). The pcDNA-cKIT construct was synthesized by Miaoling biotechnology (Wuhan, China).

HEK293T cells were obtained from Shanghai Institute of Biochemistry and Cell Biology, Chinese Academy of Science and maintained in high-glucose Dulbecco’s modified Eagle’s medium (DMEM, 10013-CV, Corning Incorporated, Corning, NY, USA USA) supplemented with 10% fetal bovine serum (FBS, TIANHANG Biotechnology Company, Huzhou, China) and antibiotics (100 units/mL penicillin and 100 μg/mL streptomycin, Amresco, Solon, OH, USA) in a 37 °C incubator with 5% CO_2_.

### 4.3. Mice

Male C57BL/6 mice (6–8-week-old) were purchased from Liaoning Changsheng biotechnology (Shenyang, China). Mice were fed with a normal diet and maintained in specific-pathogen-free (SPF) and temperature- and light-controlled conditions. All the experimental procedures conformed to the Laboratory Animal Guideline for Ethical Review of Animal Welfare and were approved by the Animal Ethics Board of Northeast Normal University.

### 4.4. Generation of STS1 and STS2 HP Domain Recombinant Proteins

STS1 and STS2 HP recombinant proteins were generated by the procaryotic expression system. Briefly, an overnight culture of BL21 cells transformed with pNIC28-Bsa4-STS1-HP or pNIC28-Bsa4-STS2-HP plasmids was reinoculated at 1:50 into fresh luria-bertani (LB) medium containing 100 μg/mL ampicillin and continued to cultivate for 4 h. Then the bacterial solutions were induced with 1.0 mM (for STS1) or 0.1 mM (for STS2) isopropyl-beta-D-thiogalactopyranoside (IPTG) at 37 °C for 4 h. The cell pellets were collected by centrifugation and resuspended with resuspension buffer (50 mM NaCl, 20 mM Tris-HCl, 5 mM imidazole, pH 7.9), followed by ultrasonic disruption. The recombinant STS1/STS2 proteins in the supernatant were purified by Ni Sepharose high performance (GE Healthycare Chicago, IL, USA). The eluted recombinant STS1/STS2 proteins were desalted and concentrated by an ultrafiltration tube.

### 4.5. Enzyme Kinetic Analysis

The phosphatase activities of STS1 and STS2 proteins were determined by an in vitro phosphatase assay using pNPP (p7998, Sigma-Aldrich, USA) as the substrate. pNPP, STS1 and STS2 proteins were prepared with reaction buffer (25 mM HEPES, 50 mM NaCl, 5 mM dithiothreitol and 2.5 mM EDTA, pH 7.2). Then 50 μL of pNPP and 10 μL of STS1 (150 nM) or STS2 (15 μM) were added into a 96-well plate to initiate the reaction at 25 °C. A total of 40 μL of NaOH stop buffer (final concentration of 0.15 M) was added to stop the reaction after 10 min (for STS1) or 20 min (for STS2), and the absorbance of the reaction product *p*-nitrophenol (pNP) at 405 nm was measured on SpectraMax Plus (Molecular Devices, Sunnyvale, CA, USA). The velocities were calculated with the equation shown as follows:

Velocity (mM/min) = OD405nm/time/light path (cm)/molar extinction coefficient *(1)

* Molar extinction coefficient for pNP is 18 mM^−1^ cm^−1^ [62].

The velocities at different substrate concentrations were plotted against the corresponding substrate concentrations. The Michaelis-Menten equation was fit using Graphpad Prism 8.0 software to determine the K_m_ and K_cat_ values.

### 4.6. Screening for STS1/STS2 Phosphatase Inhibitors

A total of 50 μL of pNPP together with 3 μL of 0.2 mg/mL compound, negative control solvent (20% DMSO/80% saline solution) or positive control (5 mM Na_3_VO_4_), respectively, was added to the wells. Then, 10 μL of STS1 (150 nM) or STS2 (15 μM) was added. All components were mixed well and incubated at 25 °C for 10 min (STS1) or 20 min (STS2), followed by the addition of 40 μL of stop buffer. The absorbance values of reaction products were measured on SpectraMax Plus. Every group set 6 parallels, and the inhibition ratios were calculated according to the following equation:

Inhibition ratio (%) = (1-OD405nm value of compound group/OD405nm value of negative group) × 100%(2)

### 4.7. Molecular Docking

The crystal structures of STS1 (ID: 5W5G) and STS2 (ID: 5WDI) HP domains downloaded from RCSB PDB were selected as the receptors. The BC (CID: 5282605) structure in the PubChem database was used as the ligand. First, the ligand co-crystalized with the STS1 or STS2 HP domain was withdrawn, and then the hydrogen atoms were added and water molecules in the STS1 or STS2 HP domain were removed simultaneously. The active sites of STS1 or STS2 were defined as the residues within 6 Å of the ligand in the X-ray structures. The genetic algorithm parameters of the ligands were set as 30, and the early termination command was turned off. Then BC was redocked with the proteins. SYBYL-X was used to generate potential three-dimensional conformation of BC, and GOLD 5.2 was used for molecular docking [63].

### 4.8. Capillary Electrophoresis Assay

A capillary electrophoresis assay was performed as previously described [64,65]. Then, 1 mg of BC was dissolved in 300 μL of DMSO and added to 9700 μL of 20 mM phosphate buffered saline (PBS), pH 7.0. STS1 and STS2 were detected by UV at 230 nm wavelength. Next, 0.1 M NaOH for 5 min, distilled water for 2 min and PBS for 5 min were explored successively to pretreat the capillary before electrophoresis or rinse the capillary at each electrophoresis interval. After the injection of the sample solution, 20 kV potential was applied for separation. The change of migration time was determined by the appearance of the protein peak.

### 4.9. Plasmid Transfection and Western Blot

HEK293K cells were seeded in 6-well plates and were transfected with plasmids using lipofectamine 2000 (Invitrogen, CA, USA) according to the manufacturer’s instructions when the cell confluency reached around 70%. Then, 48 h after transfection, the cell culture medium was replaced with the serum-free medium with 10 μg/mL BC. After BC treatment for different periods of time, the cells were lysed immediately and the whole cell lysates were subjected to SDS-PAGE and Western blotting as previously described [64].

### 4.10. Mouse BMMNC Isolation and In Vitro Culture

Mice were sacrificed by cervical dislocation and immersed in 75% ethanol for 15 min. Bilateral tibias and femurs were collected, and the marrow cavity was exposed by scissors. The bone marrow (BM) cells were flushed with 3 mL of prechilled PBS per bone and filtered with a 70 μM cell strainer. BMMNCs were obtained after the red blood cells (RBC) in cell suspension were lysed with ammonium chloride solution (StemCell Tecnologies, Canada). In total, 1 × 10^7^ BMMNCs were seeded in 6-well plates and cultured in 2 mL of Iscove’s Modified Dulbecco Medium (IMDM, Thermo Fisher Scientific, Waltham, MA, USA) supplemented with 10% FBS (Ausbian, Australia), murine SCF (100 ng/mL), murine TPO (50 ng/mL) and murine IL-3 (25 ng/mL) in a 37 °C incubator with 5% CO_2_.

### 4.11. Human CD34^+^ Cord Blood Cell Isolation and In Vitro Culture

Human umbilical cord blood samples were provided by the Second Hospital of Jilin University from healthy parturients with informed consent and were approved by the Ethics Committee of the Second Hospital of Jilin University. The umbilical cord blood was collected into heparin sodium blood collection bags, stored at 4 °C and sorted within 24 h. Human CD34^+^ cord blood cells were pre-enriched and sorted using EasySep^TM^ Human Cord Blood CD34 Positive Selection Kit Ⅱ (StemCell Technologies, Vancouver, BC, Canada), and all procedures were carried out in strict accordance with the manufacturer’s instructions. In total, 1 × 10^5^ CD34^+^ cells were seeded in 12-well plates and maintained in 1 mL of StemSpan^TM^ SFEM Ⅱ (StemCell Technologies, Vancouver, BC, Canada) serum-free medium supplemented with human SCF (100 ng/mL), human TPO (100 ng/mL) and human IL-6 (100 ng/mL) in a 37 °C incubator with 5% CO_2_.

### 4.12. Spleen Cell Isolation

The fresh mouse spleen was minced into small pieces with a razor and further crushed in prechilled PBS using a tissue grinder. The suspension with crushed spleen was filtered through 200-mesh copper grids and then incubated in ammonium chloride solution to lyse the red blood cells. The cells were then collected by centrifugation, washed with PBS and resuspended in PBS for future use.

### 4.13. Flow Cytometry Analysis

For photo-flow analysis, mouse BMMNCs or human CD34^+^ cord blood cells were stimulated with 10 μg/mL BC for different periods of time (2 min, 5 min, 10 min and 15 min); thereafter, cells were immediately fixed with Cytofix/Cytoperm (BD Biosciences) at 4 °C for 30 min in the dark and permeabilized with Perm/Wash buffer (BD Biosciences San Jose, CA, USA). Then cells were stained with indicated primary antibodies or isotype controls at 4 °C for 30 min in the dark and incubated with corresponding fluorescent secondary antibodies for 10 min at room temperature. Cells were washed with PBS and resuspended in 200 μL of PBS before flow cytometry analysis using a CytoFLEX S Flow Cytometer (Beckman Coulter, Miami, FL, USA). Data were analyzed with FlowJo 10.0 software.

For mouse HSPC phenotype analysis, the LSK population was defined as Lineage-Sca-1^+^cKIT^+^, long-term and short-term hematopoietic stem cells (LT- and ST-HSCs) were defined as CD34^−^FLT3^−^ and CD34^+^FLT3^−^ within the LSK population, respectively, and multipotent progenitors (MPPs) were defined as CD34^+^FLT3^+^ within the LSK population.

For human HSC phenotype analysis, primary HSCs were defined as CD34^+^CD38^−^ CD45RA^−^CD90^+^, and HSPCs were defined as CD34^+^CD38^−^.

For T and B lymphocyte analysis in mouse spleen, spleen cells were stained with antibodies against CD3-FITC to define T cells and CD19-APC to define B cells, respectively.

### 4.14. Animal Experiments

To examine the effects of BC treatment on healthy bone marrow in mice, C57BL/6 mice were given by intraperitoneal (i.p.) injection BC (10 or 20 mg/kg) or the same volume of solvent (vehicle control) for 7 days (once a day) and sacrificed 24 h after the last administration of BC.

To assess the effects of BC treatment on myelosuppression in mice, 5-FU (Sigma Aldrich, St. Louis, MO, USA) was used to induce myelosuppression in mice. Lethal doses of 5-FU (150 mg/kg) dissolved in saline were i.p. injected into mice every 7 days, and around 4 weeks, the mice would die from myelosuppression. For survival analysis, BC at indicated doses was either administered by i.p. injection once a day for 3 consecutive days before the first injection of 5-FU to test the preventive effect, or administered by i.p. injection immediately after each 5-FU injection, or administered by i.p. injection once 24 h after the first 5-FU injection, to test the therapeutic effects of BC. The survival was monitored over the experiment. To analyze the alleviation effect of BC on myelosuppression, BC was administered by i.p. injection once a day for 3 consecutive days before the first injection of 5-FU, and mice were sacrificed 7 days after the second 5-FU injection, followed by BMMNC and spleen isolation and analysis.

BC used for the above animal experiments was prepared as follows: BC was dissolved in DMSO to obtain a 0.25 mg/mL stock solution, which was stored at −20 °C before use. A certain volume of BC stock solution according to the indicated doses was first mixed well with the same volume of Tween-80, which was then dissolved in 200 μL of saline for one mouse injection.

### 4.15. CFU Assay

In total, 1 × 10^4^ mouse BMMNCs which were cultured in vitro with or without BC treatment (20 μg/mL) for 4 days or 2 × 10^3^ freshly isolated bone marrow cells from mice were plated in 1 mL of MethoCult^TM^ GF M3434 (StemCell Tecnologies, Vancouver, BC, Canada) and cultured in a 37 °C incubator with 5% CO_2_ for 12 days, while 2 × 10^3^ human CD34^+^ cord blood cells which were cultured in vitro with or without BC (10 μg/mL) for 7 days were plated in 1 mL of MethoCult^TM^ H4034 optimum (StemCell Tecnologies, Vancouver, BC, Canada) and cultured in a 37 °C incubator with 5 °C for 12 days.

All assays were carried out in 6-well plates in triplicate following the manufacturer’s instructions. CFU were scored, and colony numbers were counted under an optical microscope (Olympus, Tokyo, Japan).

### 4.16. Histological Analysis

Spleens and unilateral femurs that were dissected from mice were fixed in 10% neutral buffered formalin for 24 h and 12 h, respectively. Then spleens were dehydrated with alcohol solution and embedded with paraffin, and after dewaxing with xylene, spleens were cut into 5 μm thick sections and stained with hematoxylin and eosin (HE) as previously described [66].

Fixed femurs were decalcified first with 10% ethylene diamine tetraacetic acid (EDTA) and then washed with flowing water overnight. The decalcified bones were dehydrated, embedded, dewaxed sequentially and, finally, subjected to HE staining.

### 4.17. Peripheral Blood Item Analysis

Peripheral blood from mice was collected into an EDTA anticoagulant tube. The WBC, RBC and PLT counts were determined using a Mindary BC-10 hematology analyzer (Mindary, Shenzhen, China).

### 4.18. Statistical Analysis

In this study, all statistical analyses were conducted using SPSS 19.0 and GraphPad Prism 8.0 software. Comparisons of differences between groups were performed by Student’s *t* test or one-way ANOVA. All experiments were independently repeated at least three times, and the data were presented as the mean ± standard error of mean.

## Figures and Tables

**Table 1:** Kinetic constants of STS proteins.

Enzyme	Km (mM)	K_cat_ (s^−1^)	k_cat_/Km (M^−1^ s^−1^)
STS1-HP	3.52 ± 0.44	67.7 ± 1.31	(1.95 ± 0.21) × 10^4^
STS2-HP	5.39 ± 0.52	0.27 ± 0.005	50.51 ± 4.51

**Table 2:** Docking scoring function of baicalein with STS1 and STS2 HP domains.

	Goldscore	ASP
BC lock with STS1-HP	51.64	9.82
BC lock with STS2-HP	54.33	37.44

## Data Availability

The data presented in this study are available on request from the corresponding author.

## Supplementary Materials

The following supporting information can be downloaded at: https://www.mdpi.com/article/10.3390/ijms24032987/s1.
