# Supplementary material for: STS1 and STS2 Phosphatase Inhibitor Baicalein Enhances the Expansion of Hematopoietic and Progenitor Stem Cells and Alleviates 5-Fluorouracil-Induced Myelosuppression
Source: Int J Mol Sci. 2023 Feb 3;24(3):2987. doi: 10.3390/ijms24032987 (PMC9917816; doi:10.3390/ijms24032987)
Supplement: Supplementary file 1 [file ijms-24-02987-s001.zip › ijms-2094195-supplementary.pdf]

Supplementary Figure S1

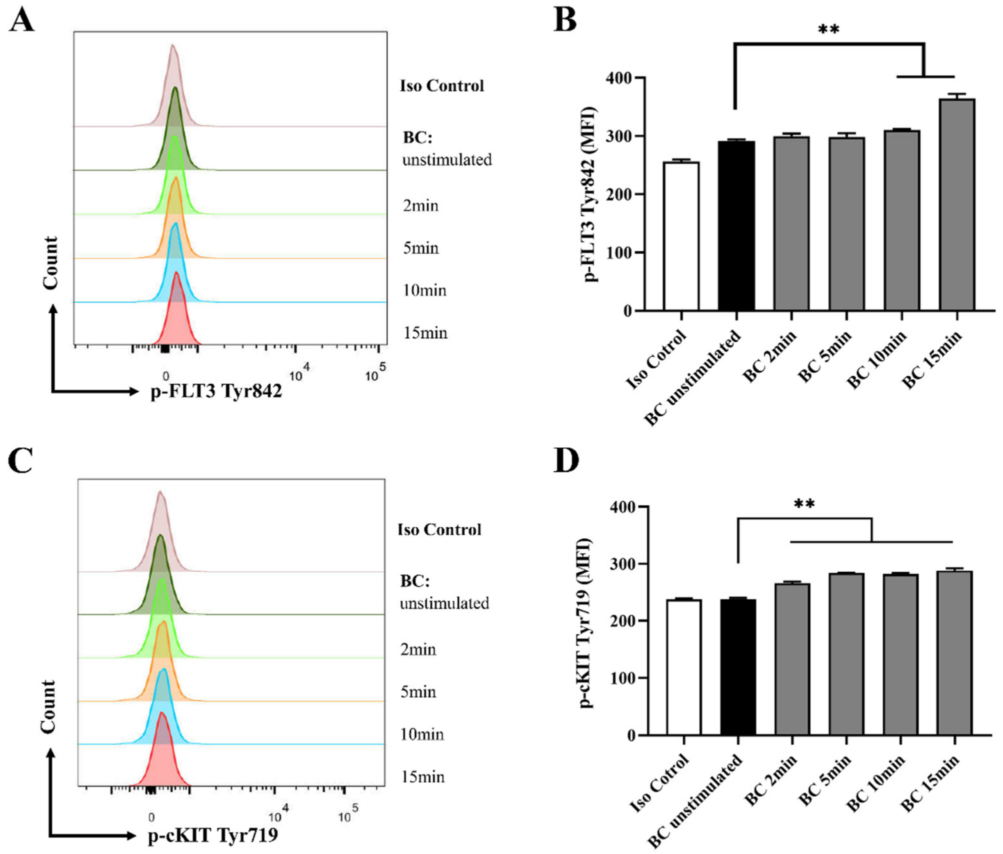

Supplementary Figure S1. The effects of BC on the phosphorylation of FLT3 at Tyr842 and cKIT at Tyr719 in mouse BMMNCs cultured *in vitro*. (A,C) Representative flow cytometry histogram showing the intensity of p-FLT3 Tyr842 (A) and p-cKIT Tyr719 (C) versus cell counts in BMMNCs cultured *in vitro* with or without BC treatment for different times(n=3). (B,D) MFI values of p-FLT3 Tyr842 (B) and p-cKIT Tyr719 (D) detected by flow cytometry in (A) and (C) (n=3). All data are shown as mean ± SEM. ** $p < 0.01$ (Student's t test).

Supplementary Figure S2

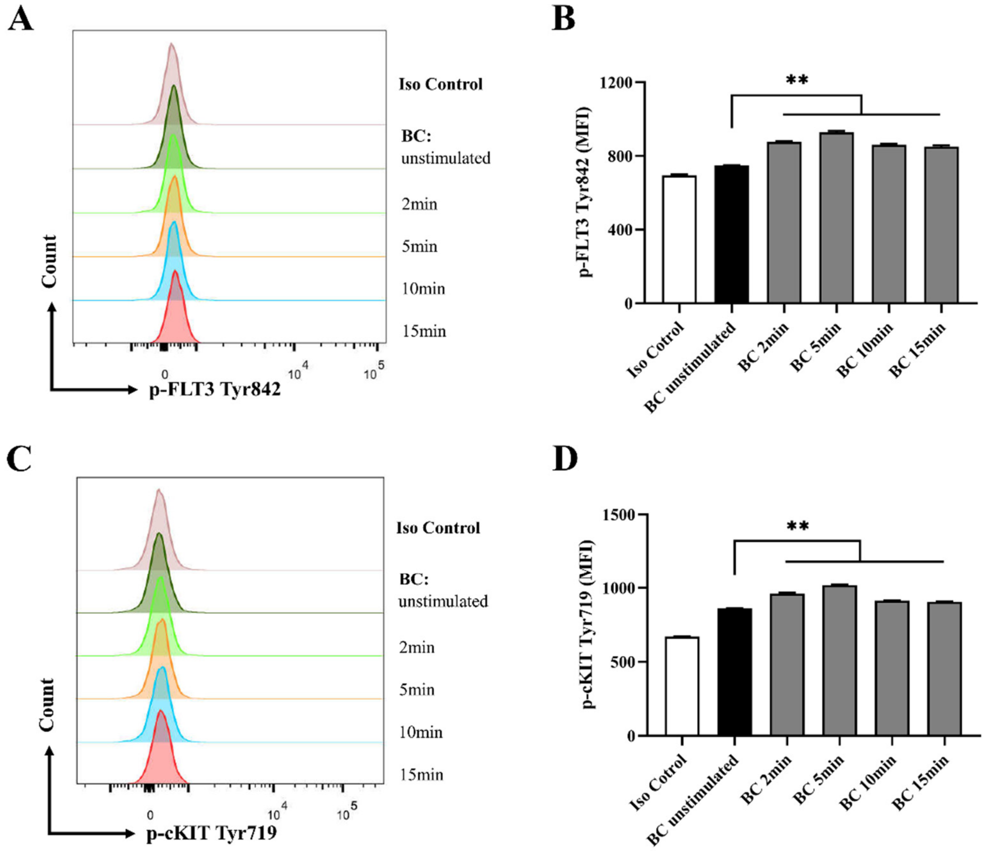

Supplementary Figure S2. The effects of BC on the phosphorylation of FLT3 at Tyr842 and cKIT at Tyr719 in human CD34⁺ UCB cultured *in vitro*. (A,C) Representative flow cytometry histogram showing the intensity of p-FLT3 Tyr842 (A) and p-cKIT Tyr719 (C) versus cell counts in human CD34⁺ UCB cultured *in vitro* with or without BC treatment for different times(n=3). (B,D) MFI values of p-FLT3 Tyr842 (B) and p-cKIT Tyr719 (D) detected by flow cytometry in (A) and (C) (n=3). All data are shown as mean ± SEM. ** $p < 0.01$ (Student's t test).

Supplementary Figure S3

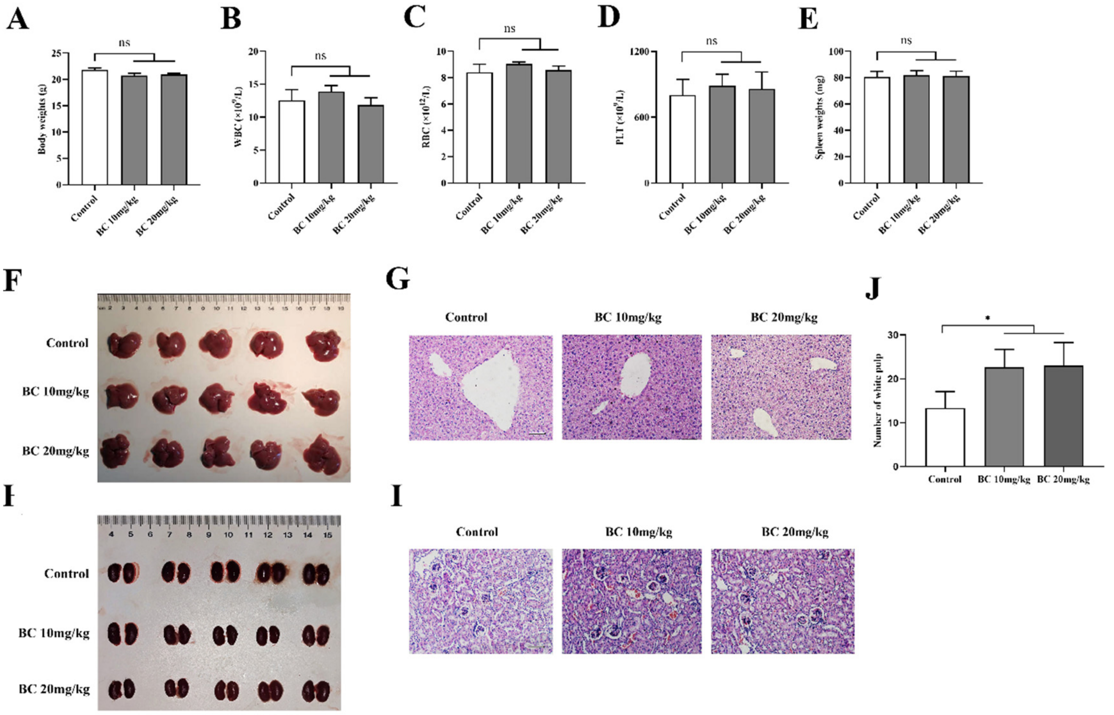

Supplementary Figure S3. The influence of BC administration for consecutive 7 days on periphery blood parameters, spleen and metabolic organs in healthy mice. C57BL/6 mice were i.p. injected with vehicle or BC (10 or 20 mg/kg) for 7 days (once a day) and then sacrificed 24 hours after last injection. (A) Body weights (*n*=5). (B-D) WBC (B), RBC (C) and PLT (D) counts in mouse periphery blood (*n*=5). (E) spleen weights (*n*=5). (F-I) Gross picture and histological analysis by HE staining of liver (F and G) and kidney (H and I) tissues. Scale bar: 50 μm. (J) Quantitation analysis of white pulp number of spleen in figure 5B (*n*=3). All data error bars are shown as mean ± SEM, ns, no significance (Student's *t* test).

Supplementary Figure S4

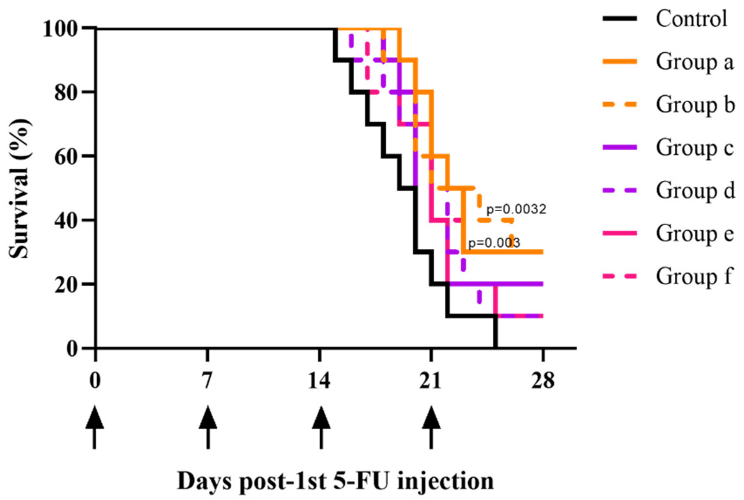

Supplementary Figure S4. The effects of BC administration on the survival of mice with 5-FU cycle injection. Control: mice were i.p. injected with saline once a day for 3 consecutive days and 24h after the last injection, mice were subjected to 5-FU injection. Group a and b: mice were i.p. injected with 10 mg/kg (a) or 20 mg/kg (b) BC once a day for 3 consecutive days and 24h after the last injection, mice were subjected to 5-FU injection. Group c and d: mice were i.p injected with 10 mg/kg (c) or 20 mg/kg (d) BC once, simultaneously with the first injection of 5-FU. Group e and f: mice were i.p. injected with 10 mg/kg (e) or 20 mg/kg (f) BC once, 24h after the first 5-FU injection. For 5-FU injection, 150mg/kg 5-FU were i.p. injected once every 7 days (arrows indicate the day of injection). 12 mice per group.
